# The *Arabidopsis* MIEL1 E3 ligase negatively regulates ABA signalling by promoting protein turnover of MYB96

**DOI:** 10.1038/ncomms12525

**Published:** 2016-09-12

**Authors:** Hong Gil Lee, Pil Joon Seo

**Affiliations:** 1 Department of Biological Sciences, Sungkyunkwan University, Suwon 16419, Republic of Korea

## Abstract

The phytohormone abscisic acid (ABA) regulates plant responses to various environmental challenges. Controlled protein turnover is an important component of ABA signalling. Here we show that the RING-type E3 ligase MYB30-INTERACTING E3 LIGASE 1 (MIEL1) regulates ABA sensitivity by promoting MYB96 turnover in *Arabidopsis*. Germination of *MIEL1*-deficient mutant seeds is hypersensitive to ABA, whereas *MIEL1*-overexpressing transgenic seeds are less sensitive. MIEL1 can interact with MYB96, a regulator of ABA signalling, and stimulate its ubiquitination and degradation. Genetic analysis shows that *MYB96* is epistatic to *MIEL1* in the control of ABA sensitivity in seeds. While MIEL1 acts primarily via MYB96 in seed germination, MIEL1 regulates protein turnover of both MYB96 and MYB30 in vegetative tissues. We find that ABA regulates the expression of MYB30-responsive genes during pathogen infection and this regulation is partly dependent on MIEL1. These results suggest that MIEL1 may facilitate crosstalk between ABA and biotic stress signalling.

## Abstract (web summary)

The phytohormone abscisic acid controls plant responses to environmental stress, partly by regulating protein turnover. Here the authors propose that abscisic acid regulates seed germination by promoting degradation of the MYB96 transcription factor via the MIEL1 E3 ubiquitin (Ub) ligase.

## Graphical abstract

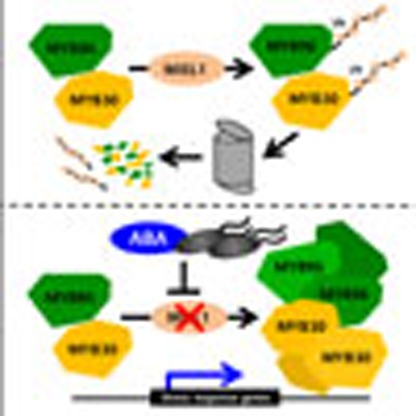

As sessile organisms, plants have evolved sophisticated biochemical and physiological responses to deal with environmental challenges, such as drought, high salinity, wounding and temperature extremes^1^. The phytohormone ABA contributes in part to plant adaptation to environmental fluctuations by modulating a wide array of physiological processes, including seed dormancy and germination, early seedling growth, guard cell functioning and stress tolerance^2^,^3^,^4^,^5^.

ABA is perceived by the receptor proteins PYRABACTIN RESISTANCE 1 (PYR)/PYR1-LIKE (PYL)/REGULATORY COMPONENTS OF ABA RECEPTOR^6^,^7^. In the presence of ABA, these receptors interact with protein phosphatase 2C (PP2C) proteins such as ABA-INSENSITIVE 1 (ABI1) and ABI2, which negatively regulate ABA signalling, and inhibit their catalytic activities^8^. Subsequently, SNF1-RELATED KINASE 2 s are derepressed from PP2Cs and phosphorylate leucine-zipper ABA-responsive element (ABRE)-binding proteins/ABRE-binding factors (AREBs/ABFs), which bind directly to ABREs of stress-responsive genes, to stimulate their transcriptional activities^8^,^9^.

In addition to AREBs/ABFs, MYB and MYC transcription factors also constitute ABA-dependent signalling pathways. MYB2 and MYC2 cooperatively regulate the drought-responsive *RESPONSIVE TO DESSICATION 22* (*RD22*) gene by directly binding to its promoter^10^,^11^. Several R2R3-type MYB transcription factors, including MYB15, MYB20, MYB41, MYB44 and MYB96, also mediate ABA signalling and regulate a variety of physiological responses under stress conditions, such as root and shoot development, hormone metabolism, cell expansion, drought tolerance and cuticular wax biosynthesis^12^,^13^,^14^,^15^,^16^,^17^,^18^, underscoring the importance of transcriptional regulation in plant stress responses.

Transcription factor activities are further shaped by additional post-translational modifications. In particular, the ubiquitination process is a representative way of modulating protein turnover and is mediated by the sequential action of three enzymes: Ub-activating enzyme (E1), Ub-conjugating enzyme (E2) and Ub ligase (E3)^19^,^20^,^21^. Notably, the *Arabidopsis* genome encodes >1,500 E3 enzymes^19^,^20^, and this large number of E3 ligases implies specific recognition of target substrates^22^.

Among the *Arabidopsis* E3 ligases, >470 proteins belong to the RING-finger protein family^20^,^23^. Remarkably, a significant number of the members are responsible for ABA signal transduction^22^,^24^,^25^. For instance, the RING-type E3 ligase RING FINGER OF SEED LONGEVITY 1 stimulates protein turnover of PYR4 and PYR1 at the plasma membrane^26^. RING-type E3 ligase ABI3 INTERACTION PROTEIN 2 (AIP2) interacts with and targets ABI3 for protein degradation^27^. ABI5 is also proteolytically degraded by RING-finger protein KEEP ON GOING, ensuring a low level of ABI5 production in the absence of ABA^28^,^29^. Protein stability of ABA-inducible DEHYDRATION-RESPONSIVE ELEMENT BINDING PROTEIN2A (DREB2A) is also controlled by C3HC4 RING-type E3 ligases DREB2A-INTERACTING PROTEIN 1 (DRIP1) and DRIP2 (ref. 30). In addition, the SALT- AND DROUGHT-INDUCED RING FINGER 1 is a positive regulator of ABA signalling in seed germination, stomatal closure and drought tolerance^31^. The RING-H2 E3 ligases RHA2a and RHA2b also regulate ABA-dependent seed germination, early seedling development and drought tolerance through an ABI3/4/5-independent pathway^32^,^33^.

In this study, we demonstrate that the RING-type E3 ligase MIEL1, which was previously reported as a negative regulator of hypersensitive cell death^34^, is involved in ABA regulation of seed germination by promoting MYB96 turnover in *Arabidopsis*. We found that *MIEL1*-deficient mutant seeds are hypersensitive to ABA, whereas *MIEL1*-overexpressing transgenic seeds (35S:*MIEL1-MYC*) exhibit a hyposensitive response during the germination. The MIEL1 function in ABA-modulated seed germination depends primarily on MYB96. However, in vegetative stages, MIEL1 triggers simultaneous protein turnover of MYB96 and MYB30, and we show that ABA can influence expression of MYB30-regulated genes related to biotic stress. We propose that MIEL1 coordinates ABA-dependent adaptive responses and defensive hypersensitive responses in *Arabidopsis*.

## Results

### MIEL1 regulates ABA-dependent seed germination

Ubiquitination is a representative molecular mechanism that regulates ABA signalling^20^,^22^,^24^. To further understand the ABA signalling network, we analysed the ABA responsiveness of several E3 ligase mutant seeds to identify additional regulator(s) of the process^35^.

**Figure 1:**
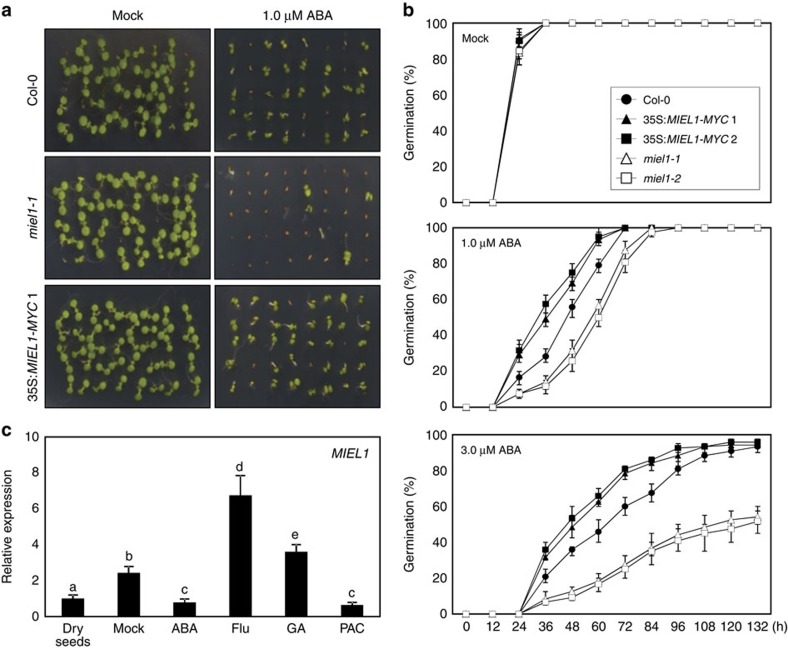
ABA sensitivity of 35S:*MIEL1-MYC* transgenic and *miel1*-deficient seedlings during germination. (**a**) Altered ABA sensitivity of 35S:*MIEL1-MYC* and *miel1-1* early seedlings. Seeds were germinated and grown on 1 μM ABA under long-day (LD) conditions. Photographs were taken 3 days after cold stratification. (**b**) Germination percentage. The percentage of seed germination of the indicated genotypes grown on different concentrations of ABA was quantified after the end of stratification. Radicle emergence was used as a morphological marker for germination. At least 50 seeds per genotype were measured in each replicate. Biological triplicates were averaged. Bars indicate the s.e.m. (**c**) *MIEL1* expression in seeds. Seeds were germinated on MS medium supplemented with 1 μM ABA, 10 μM fluridone (Flu), 0.5 μM GA or 15 μM paclobutrazol (PAC) and incubated for 2 days under LD conditions. Transcript accumulation was analysed by RT–qPCR. Three independent biological replicates were averaged. Different letters represent a significant difference at *P*<0.05 (one-way analysis of variance with Fisher's *post hoc* test). Bars indicate the s.e.m.

Among the mutants examined, we preliminarily found that *miel1-1* seeds were hypersensitive to ABA during germination (Fig. 1a). To validate the results of our screen, we obtained an additional mutant allele *miel1-2* and also generated *MIEL1*-overexpressing 35S:*MIEL1-MYC* transgenic plants (Supplementary Fig. 1). The germination kinetics of the *mie11* mutant and 35S:*MIEL1-MYC* transgenic seeds were comparable to that of wild-type seeds in the absence of ABA (Fig. 1b; Supplementary Fig. 2). However, in the presence of ABA, germination of *miel1-1* and *miel1-2* mutant seeds was significantly delayed, whereas the 35S:*MIEL1-MYC* transgenic seeds exhibited reduced ABA sensitivity (Fig. 1b; Supplementary Fig. 2). The differences in the germination timing of the *MIEL1*-deficient mutant and 35S:*MIEL1-MYC* transgenic seeds were maintained at higher ABA concentrations (Fig. 1b; Supplementary Fig. 2). Although the impact of MIEL1 in ABA-dependent seed germination was not strong as much as that of ABI1, its role in seed germination was still relevant and significant (Supplementary Fig. 2).

To support the roles of MIEL1 in seed germination, we analysed the transcript accumulation of *MIEL1* in seeds. Expression of *MIEL1* in dry seeds was comparable to that in 7-day-old seedlings (Supplementary Fig. 3). In addition, transcript accumulation of *MIEL1* was substantially elevated upon the imbibition and stratification (Supplementary Fig. 3). Furthermore, *MIEL1* was induced by gibberellic acid (GA) and the ABA synthesis inhibitor fluridone, which accelerate seed germination, whereas ABA and the GA synthesis inhibitor paclobutrazol suppressed *MIEL1* in seeds (Fig. 1c). These observations are consistent with public expression data (eFP browser)^36^, which shows that the *MIEL1* gene is strongly expressed in micropylar endosperm (Supplementary Fig. 4). The micropylar endosperm surrounding the radicle tip acts as a physical barrier of seed germination and is the major place, where antagonistic actions of ABA and GA (or ethylene) are supposed to regulate endosperm weakening and thus endosperm rupture/radicle protrusion^36^,^37^,^38^. Given that the 26S proteasome activity is required for micropylar endosperm autolysis^39^, MIEL1 might play a role in this process.

### MIEL1 can interact with MYB96

**Figure 2:**
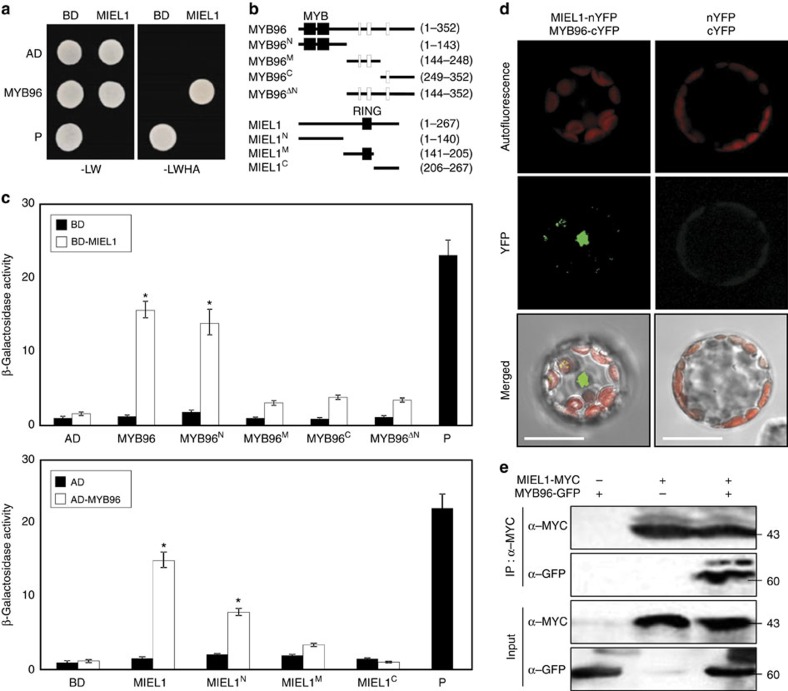
Interaction of MIEL1 with MYB96. (**a**) Yeast-two-hybrid (Y2H) assays. Y2H assays were performed with the MIEL1 protein fused with the DNA-binding domain (BD) of GAL4 and MYB96 fused with the transcriptional activation domain (AD) of GAL4 for the analysis of their interactions. Interactions were examined by the cell growth on selective media. -LWHA indicates Leu, Trp, His and Ade drop-out plates. -LW indicates Leu and Trp drop-out plates. GAL4 was used as a positive control (P). (**b**) Deletion constructs of MIEL1 and MYB96. Numbers indicate residue positions. (**c**) Interaction domain mapping. β-Galactosidase (β-Gal) activity was quantified after growing yeast strains in liquid culture with *o*-nitrophenyl-β-D-galactopyranoside as a substrate. Three independent measurements of β-Gal activities were averaged and statistically analysed by a Student's *t*-test (*P<0.05). Bars indicate the s.e.m. (**d**) BiFC assays. Partial YFP fusion constructs containing either MIEL1 or MYB96 were transiently co-expressed in *Arabidopsis* protoplasts. Chloroplasts appear in red. Scale bars, 20 μm. (**e**) Co-immunoprecipitation assays. *A. tumefaciens* cells containing MIEL1-MYC and MYB96-GFP constructs were injected to 3-week-old *N. benthamiana* leaves. Epitope-tagged proteins were detected immunologically using corresponding antibodies. The molecular weight (kDa) was indicated on the right side of the gel. IP, immunoprecipitation.

MIEL1 is an E3 Ub ligase that commonly facilitates protein degradation through physical interactions with a target substrate^34^. Therefore, we asked which protein might be a regulatory target of MIEL1 in the regulation of ABA sensitivity. We performed a yeast-two-hybrid (Y2H) screen and identified a physical interaction with a partial fragment of MYB96, a key mediator of ABA signalling in a variety of physiological processes^15^,^16^,^17^,^40^,^41^,^42^. To validate this observation, the full-size *MYB96* gene was fused in-frame to the 3′-end of activation domain (AD) of GAL4, and the construct was co-expressed with GAL4 DNA-binding domain (BD)-MIEL1 fusion construct in yeast cells. As expected, MYB96 interacted with MIEL1 in yeast cells (Fig. 2a).

To identify the regions responsible for the interaction between MIEL1 and MYB96, we constructed a series of deletion forms of these two proteins (Fig. 2b). Y2H analysis revealed that the N-terminal region of MYB96 (residues 1–143) was responsible for the interaction between MIEL1 and MYB96. Likewise, MYB96 specifically interacted with the N-terminal region of MIEL1 (residues 1–140; Fig. 2c).

To further support the interaction of MIEL1 with MYB96 *in vivo*, we also performed bimolecular fluorescent complementation (BiFC) analysis using *Arabidopsis* protoplasts. The *MYB96* complementary DNA (cDNA) sequence was fused in-frame to the 5′-end of a gene sequence encoding the C-terminal half of YFP, and the *MIEL1* gene was fused in-frame to the 5′-end of a sequence encoding the N-terminal half of YFP. The fusion constructs were then transiently co-expressed in *Arabidopsis* protoplasts. Yellow fluorescence was exclusively detected in the nucleus (Fig. 2d), while co-expression with an empty vector did not show any discernible fluorescence (Fig. 2d; Supplementary Fig. 5). The *in planta* interaction of MIEL1 and MYB96 was also verified by co-immunoprecipitation (Co-IP) assay using *Nicotiana benthamiana* cells transiently co-expressing MIEL1-MYC and MYB96-GFP fusion constructs (Fig. 2e).

We then asked whether the MIEL1-MYB96 interactions are mutually specific in the context of ABA signalling components. Several E3 Ub ligases responsible for ABA signal transduction, including AIP2, ATL43, ARM REPEAT PROTEIN INTERACTING WITH ABF2, CARBOXYL TERMINUS OF HSC70-INTERACTING PROTEIN and XERICO^43^, were employed to generate GAL4 BD fusion constructs, and the fusion constructs were used to coexpress with the GAL4 AD-MYB96 fusion construct in yeast cells. No discernible interaction was observed among the diverse combinations (Supplementary Fig. 6). Furthermore, MIEL1 was primarily associated with MYB96, and other transcriptional regulators were not preferred as interactive targets (Supplementary Fig. 6), suggesting a specific interaction between MIEL1 and MYB96 among components of ABA signalling networks.

### MIEL1 ubiquitinates MYB96 and facilitates its degradation

**Figure 3:**
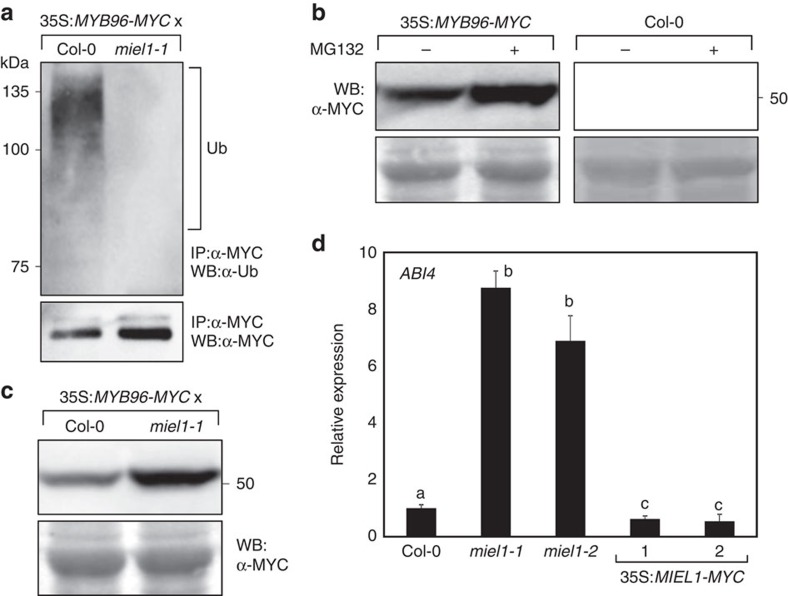
Ubiquitination-induced proteolysis of MYB96 by MIEL1. (**a**) Ubiquitination of MYB96 by MIEL1 in seeds. Stratified seeds were pretreated with 50 μM MG132 for 24 h before harvesting and used for Co-IP assays. Total protein extracts were immunoprecipitated using anti-MYC antibodies coupled to sepharose beads and were subjected to western blot analysis using anti-ubiquitin (top panel) and anti-MYC (bottom panel) antibodies. IP, immunoprecipitation; Ub, ubiquitinated MYB96 proteins; WB, western blot analysis. (**b**) Effects of MG132 on MYB96 stability. Stratified 35S:*MYB96-MYC* transgenic seeds were incubated in MS-liquid medium supplemented with or without 50 μM MG132 for 24 h. The molecular weight (kDa) was indicated on the right side of the gel. (**c**) MYB96 accumulation in *miel1-1*. Stratified seeds were used for total protein extraction. MYB96 proteins were detected immunologically using an anti-MYC antibody. (**d**) *ABI4* expression in seeds. Transcript accumulation was analysed by RT–qPCR. The *eIF4a* gene was used as an internal control. Biological triplicates were averaged. Different letters represent a significant difference at *P*<0.05 (one-way analysis of variance with Fisher's *post hoc* test). Bars indicate the s.e.m.

The physical interactions of MIEL1 with MYB96 led us to examine whether MIEL1 ubiquitinates MYB96. To examine ubiquitination of MYB96 by MIEL1 *in vivo*, we carried out Co-IP analysis. Total protein extracts from 35S:*MYB96-MYC* and 35S:*MYB96-MYC*x*miel1-1* seeds were immunoprecipitated with an anti-MYC antibody. The precipitates were subsequently used for western blot analysis with anti-Ub or anti-MYC antibody. Co-IP analysis revealed that MYB96 was subjected to ubiquitination in wild-type background, but the ubiquitination of MYB96 was substantially reduced in *miel1-1* background (Fig. 3a), supporting that MIEL1 stimulates ubiquitination of MYB96.

MYB96 ubiquitination may lead to its proteasomal degradation. To investigate this possibility, we employed MG132, a potent chemical inhibitor of the 26S proteasome, and investigated its effects on MYB96 stability. Immunoblot analysis showed that MYB96 abundance was significantly elevated upon the treatment with MG132 in seeds (Fig. 3b). For comparison, we also measured MYB96 accumulation in seedlings after MG132 application and found that ubiquitination-dependent MYB96 degradation was also relevant in seedlings (Supplementary Fig. 7). Furthermore, we analysed MYB96 accumulation in seeds and seedlings of 35S:*MYB96-MYC* and 35S:*MYB96-MYC*x*miel1-1* genotypes. As expected, MYB96 stability was increased in *miel1-1* background, regardless of plant developmental stages (Fig. 3c; Supplementary Fig. 8), even though transcript levels of *MYB96* were similar in both genotypes (Supplementary Fig. 9).

It was reported that MYB96 regulates ABA-dependent seed germination by promoting *ABI4* transcription^42^. We therefore postulated that MIEL1 might also influence *ABI4* expression in seeds, possibly through protein degradation of MYB96. Consistent with the previous observations^42^, expression of *ABI4* was upregulated in *miel1* mutant seeds, but downregulated in 35S:*MIEL1-MYC* transgenic seeds (Fig. 3d). Moreover, considering the MYB96 degradation by MIEL1 in seedlings, it was supposed that MIEL1 also affects expression of ABA- and abiotic stress-responsive genes regulated by MYB96 during vegetative stages^15^,^16^,^17^,^40^. Indeed, *HEPTAHELICAL TRANSMEMBRANE PROTEIN 1* (*HHP1*), *3-KETOACYL-COA SYNTHASE 6* (*KCS6*) and *RD22* genes, which are directly controlled by MYB96 (refs 15, 16, 40), were significantly upregulated in *miel1* mutant, but repressed in 35S:*MIEL1-MYC* transgenic seedlings (Supplementary Fig. 10), indicating that MIEL1 negatively regulates MYB96 stability by means of protein ubiquitination not only during seed germination, but also vegetative stages.

Ubiquitinated proteins are often subject to degradation by the 26S proteasome. Of its two major particles, the 20S core protease and the 19S regulatory particle (RP), the RP confers substrate specificity to the holoenzyme for their breakdown^44^. Notably, the REGULATORY PARTICLE NON-ATPASE 10 (RPN10) protein, a base component of the RP, plays a role in ABA signalling attenuation by promoting ubiquitination-derived protein turnover of ABA signalling components^45^,^46^. Thus, we asked whether the MIEL1 function is associated with RPN10. Quantitative PCR with reverse transcription (RT–qPCR) analysis showed that MYB96-regulated genes such as *ABI4*, *KCS6* and *BETA-KETOACYL REDUCTASE 1* were unaffected, or rather suppressed, in *rpn10-1* (Supplementary Fig. 11), suggesting that the MIEL1-MYB96 module is functionally independent of RPN10 and might require different member(s) of the RP for the ubiquitinated MYB96 recognition. However, we cannot completely rule out the possible association of RPN10 with MIEL1 signalling in that transcript accumulation of *MIEL1* was significantly increased in *rpn10-1*, as the *ABI5* gene was altered (Supplementary Fig. 12), and RPN10 plays a fundamental role in ABA signalling^45^.

### ABA suppresses MIEL1 but promotes MYB96 accumulation

**Figure 4:**
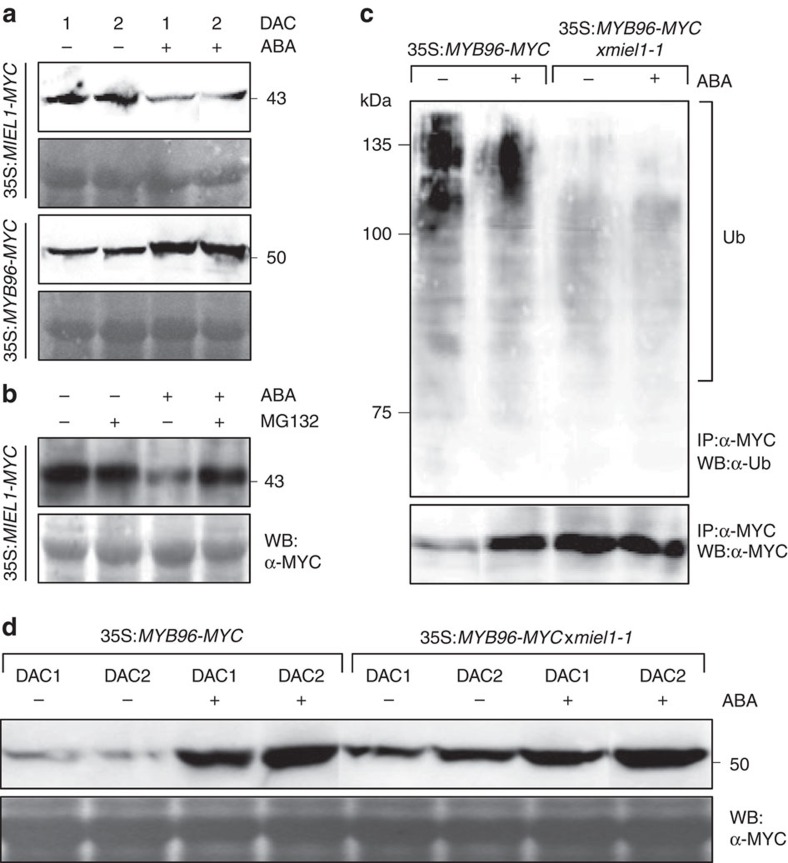
ABA regulation of MIEL1 and MYB96 stability. (**a**) Protein abundance of MIEL1 and MYB96 in the presence of ABA. Seeds were germinated on MS medium supplemented with or without 1 μM ABA and incubated for the indicated time periods after cold stratification. The molecular weight (kDa) was indicated on the right side of the gel. (**b**) Effects of MG132 on ABA-dependent MIEL1 degradation. Stratified seeds were treated with 1 μM ABA and/or 50 μM MG132 for 24 h. (**c**) Changes in ubiquitination of MYB96 in the presence of ABA. Stratified seeds were treated with 1 μM ABA for 24 h and used for the total protein extraction. (**d**) Effects of ABA on MYB96 stability in *miel1-1*. Seeds were germinated on MS medium supplemented with or without 1 μM ABA and incubated for the indicated time periods after cold stratification. DAC, days after cold stratification; IP, immunoprecipitation; Ub, ubiquitinated MYB96 proteins; WB, Western blot analysis.

The ABA sensitivity phenotype and altered accumulation of MYB96 in *miel1* mutants suggest that a MIEL1-MYB96 signalling module may be required for proper ABA signal transduction. We therefore asked how ABA regulates accumulation patterns of MIEL1 and MYB96. To address this question, we first analysed the transcript accumulation of *MIEL1*. While expression of *MIEL1* exhibited diurnal fluctuation under normal growth conditions (Supplementary Fig. 13) consistent with the light promotion of seed germination, exogenous ABA treatment significantly reduced its transcript accumulation both in seeds and seedlings (Fig. 1c; Supplementary Fig. 14). In accordance with this, protein accumulation of MIEL1 was also decreased in the presence of ABA (Fig. 4a; Supplementary Fig. 15), and its degradation was dependent on 26S proteasomal activity (Fig. 4b; Supplementary Fig. 16). Accumulation of MYB96 protein was complementary to that of MIEL1. ABA promoted MYB96 accumulation, possibly due to the derepression from the destructive activity of MIEL1 (Fig. 4a; Supplementary Fig. 15).

In support of this, ubiquitination of MYB96 was significantly reduced upon ABA treatment (Fig. 4c; Supplementary Fig. 17). The reduction of its ubiquitination was correlated to the MIEL1 activity. Ubiquitination levels of MYB96 were markedly low and insensitive to ABA in *miel1-1* background (Fig. 4c; Supplementary Fig. 17).

To further support the negative regulation of MYB96 by MIEL1 in the presence of ABA, we also examined the effects of ABA on MYB96 stability in *miel1-1* background. As expected, exogenous treatment with ABA led to the increased accumulation of MYB96 in wild-type background (Fig. 4d; Supplementary Fig. 18), whereas a high level of MYB96 accumulation was observed even in the absence of ABA and the protein levels were less sensitive to ABA treatment in the *miel1-1* mutant (Fig. 4d; Supplementary Fig. 18).

### *MYB96* is epistatic to *MIEL1* in control of seed germination

**Figure 5:**
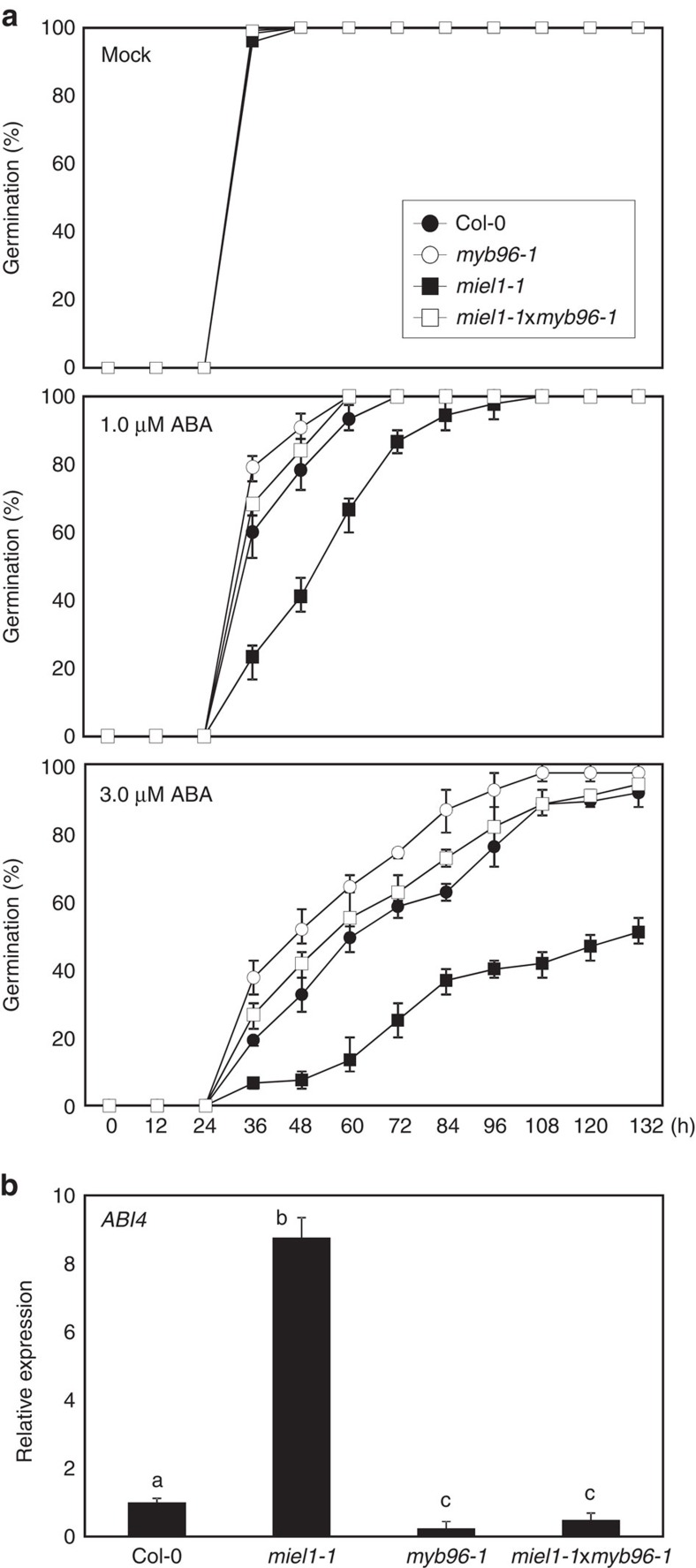
Genetic hierarchy of *MIEL1* and *MYB96* in the control of seed germination. (**a**) Germination percentage of *miel1-1*x*myb96-1*. Seed germination percentage of each genotype on different concentrations of ABA was scored at the indicated time points after cold stratification. At least 50 seeds per genotype were measured in each replicate. Biological triplicates were averaged. Bars indicate the s.e.m. (**b**) *ABI4* expression in *miel1-1*x*myb96-1*. Stratified seeds were used to analyse transcript accumulation. The *eIF4a* gene was used as an internal control. Biological triplicates were averaged. Different letters represent a significant difference at *P*<0.05 (one-way analysis of variance with Fisher's *post hoc* test). Bars indicate the s.e.m.

To confirm the genetic hierarchy between *MIEL1* and *MYB96*, we crossed the *miel1-1* mutant with *myb96-1* and measured the germination percentage. In the presence of ABA, germination of *miel1-1* seeds was significantly delayed, whereas *myb96-1* seeds exhibited reduced sensitivity to ABA compared with wild-type seeds (Fig. 5a). Notably, the germination percentage of *miel1-1*x*myb96-1* seeds was comparable to that of *myb96-1* seeds (Fig. 5a). In agreement with this, transcript accumulation of *ABI4* in *miel1-1*x*myb96-1* was also similar to that of *myb96-1* (Fig. 5b). Furthermore, no feedback regulation between *MYB96* and *MIEL1* was observed (Supplementary Fig. 19), providing further evidence that *MYB96* is epistatic to *MIEL1* and that they act in the same genetic pathway to control ABA-dependent seed germination.

### MIEL1 may facilitate ABA and defence signalling crosstalk

**Figure 6:**
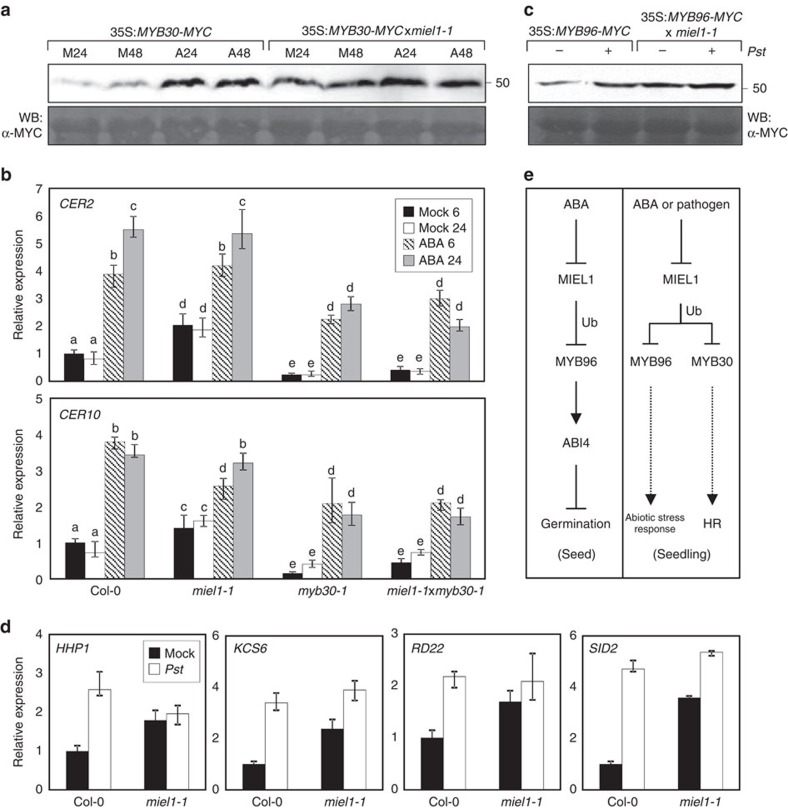
Coordinated regulation of MYB96 and MYB30 by MIEL1 in seedlings. (**a**) Effects of ABA on MYB30 stability in *miel1-1*. Two-week-old plants were incubated in MS-liquid medium supplemented with or without 20 μM ABA for the indicated time periods (h). MYB30 proteins were detected immunologically using an anti-MYC antibody. The molecular weight (kDa) was indicated on the right side of the gel. (**b**) Effects of ABA on transcript accumulation of MYB30 target genes. Two-week-old plants were incubated in MS-liquid medium supplemented with or without 20 μM ABA for the indicated time periods (h). Transcript accumulation was analysed by RT–qPCR. The *eIF4a* gene was used as an internal control. Biological triplicates were averaged. Different letters represent a significant difference at *P*<0.05 (one-way analysis of variance with Fisher's *post hoc* test). Bars indicate the s.e.m. (**c**) Effects of pathogen infection on MYB96 stability in *miel1-1*. Four-week-old plants were infected with *Pst* DC3000/*avrRpm1* (*Pst*), and the infected leaves were used for total protein isolation. (**d**) Effects of *Pst* infection on transcript accumulation of MYB96 target genes. Four-week-old plants were infected with *Pst*, and the infected leaves were used to analyse transcript accumulation. Biological triplicates were averaged. Bars indicate the s.e.m. (**e**) Proposed roles of MIEL1 in *Arabidopsis*. In seeds, MIEL1 stimulates protein turnover of MYB96, a positive regulator of ABA signalling, to promote seed germination. In the presence of ABA, MIEL1 stability is reduced, and thus MYB96 is derepressed to activate ABA signaling cascades. In seedling stages, MIEL1 may facilitate a crosstalk between abiotic and biotic signaling by regulating protein turnover of both MYB96 and MYB30, which mediate ABA-dependent drought tolerance and hypersensitive defense responses (HR), respectively. The cross-regulation probably facilitates to efficiently trigger both abiotic and biotic responses under environmentally unfavourable conditions. Ub, ubiquitination.

MIEL1 largely depends on MYB96 in the control of seed germination (Fig. 5 and see Discussion). However, given that MIEL1 additionally regulates protein stability of a hypersensitive response (HR) regulator MYB30 in response to pathogen infection^34^ and that MYB96 degradation by MIEL1 is also relevant in seedlings, MIEL1 might act in crosstalk between ABA and biotic stress signalling in vegetative tissues. To test this possibility, we examined the effects of ABA on MYB30-mediated signalling in seedlings. Notably, MYB30 turnover was suppressed by ABA in a MIEL1-dependent manner (Fig. 6a). Consistently, the *ECERIFERUM 2* (*CER2*) and *CER10* genes regulated by MYB30 during pathogen infection were considerably induced by ABA, and their expression was less sensitive to exogenous ABA treatment in the *miel1-1* mutant (Fig. 6b).

In addition, the ABA signalling component MYB96 is also associated with the plant defence against biotrophs. Wild-type plants infected with the avirulent *Pseudomonas syringae* pv. *tomato* DC3000*/avrRpm1* (*Pst* DC3000/*avrRpm1*) cells showed increased protein accumulation of MYB96 (Fig. 6c), which may be due to the reduced levels of *MIEL1* (ref. 34). MYB96 accumulation was not further responsive to pathogen infection in the *miel1-1* mutant (Fig. 6c). Consistently, MYB96-regulated ABA-inducible genes *HHP1*, *KCS6*, *RD22* and *SALICYLIC ACID INDUCTION DEFICIENT 2* were induced by *Pst* DC3000/*avrRpm1* infection, and *miel1* mutants showed constitutive high expression of them even in normal conditions (Fig. 6d).

Taken together, our findings indicate that MIEL1 is a novel ABA signalling mediator that promotes protein turnover of MYB96. This E3 Ub ligase has distinct molecular functions depending on the developmental stages. We propose that the MIEL1-MYB96 module regulates ABA signalling during seed germination, independently of MYB30 (see Discussion). In the seedling stages, we propose that MIEL1 stimulates protein turnover of both MYB96 and MYB30, which could potentially be involved in the coordination of plant responses to abiotic and biotic stresses (Fig. 6e).

## Discussion

Eukaryotic E3 Ub ligases are classified into two major groups: single-subunit E3 ligases and multi-subunit E3 ligases. Single-subunit E3 ligases include the Homologous to E6-AP Carboxyl Terminus, U-box and RING domain-containing proteins^20^,^23^. It is noteworthy that RING-finger proteins comprise the largest subgroup, and a significant number of RING proteins are involved in the control of ABA signalling (see above).

In the formation of multi-subunit E3 ligases, CULLIN (CUL) proteins act as a scaffold for assembling Ub E3 ligase complex machineries and recruit several components, including a RING-finger protein REGULATOR OF CULLINS1/RING-BOX1 and a substrate-recognition module composed of adaptor and substrate receptor proteins^23^,^47^. For example, CUL1 or CUL2 constitutes an S-PHASE KINASE-ASSOCIATED PROTEIN 1 (SKP1)-CUL-F-box-type complex along with the *Arabidopsis*-SKP1-like adaptor protein and an F-box substrate receptor protein^47^,^48^.

Multi-subunit E3 ligases also participate in ABA signal transduction. The CUL1-interacting DROUGHT TOLERANCE REPRESSOR (DOR) F-box protein mediates ABA regulation of drought stress responses^49^. The *DOR*-deficient mutants show enhanced drought tolerance with hypersensitive stomatal closure, whereas ectopic expression of *DOR* results in reduced drought tolerance.

CUL4 interacts with the DAMAGED DNA BINDING 1 (DDB1) adaptor protein and DDB1-BINDING WD40 PROTEIN (DWD)/DDB1-CUL4-ASSOCIATED FACTOR (DCAF) substrate receptor protein^49^,^50^. The *Arabidopsis* genome encodes ∼85 DWD candidates and additional 34 WDxR motif-containing proteins that do not contain an entire DWD box, but which are sufficient to interact with DDB1 (ref. 51). Notably, some of them are involved in ABA signal transduction. DWD HYPERSENSITIVE TO ABA1 (DWA1) and DWA2 regulate protein turnover of ABI5 (ref. 52). Consistently, *dwa1dwa2* double mutants exhibit altered ABA sensitivity in root growth and seed germination^52^. DWA3 is also known to negatively regulate ABA signal transduction through an ABI5-independent pathway^53^. In addition, the ABA-inducible ABA-HYPERSENSITVE DCAF1 (ABD1) protein also plays a significant role in controlled proteolysis of ABI5. The *abd1*-deficient mutants are hypersensitive to ABA in the control of seed germination, early seedling growth and drought tolerance^54^.

The CONSTITUTIVE PHOTOMORPHOGENIC10 (COP10)-DEETIOLATED1 (DET1)-DDB1 complex (CDD complex), together with a part of the substrate adaptor module DET1-, DDB1-ASSOCIATED1 (DDA1)^55^, cooperates with the CUL4-E3 ligase in the control of ABA signalling^56^. DDA1 mediates proteolytic degradation of the PYL8 ABA receptor, and DDA1 activity is attenuated in the presence of ABA^56^. Consistently, a variety of developmental processes mediated by ABA, including seed germination, early seedling growth and root growth, are altered in plants mis-expressing *DDA1* or CDD complex components^56^. Collectively, controlled proteolysis is a key molecular scheme in ABA signalling, and specific combinations of E3 ligases and ABA signalling components facilitate precise signal transduction under environmental stress conditions.

MYB96 is a representative ABA signalling mediator that regulates a variety of physiological processes, such as shoot and root development, seed germination and dormancy, cuticular wax biosynthesis, hormone homoeostasis and stress tolerance, under environmentally unfavourable conditions^15^,^16^,^17^,^40^,^41^,^42^. Considering its myriad of regulatory targets, MYB96 may act on upstream effector of the ABA signalling pathway.

Despite its importance in ABA signalling, the upstream regulators of MYB96 are largely unknown. Here we found that MIEL1 is an regulator of MYB96, which stimulates its proteolytic degradation. MIEL1 can interact with MYB96 and promote its ubiquitination. We propose that in the absence of ABA, MIEL1 accumulates and targets MYB96 for degradation to attenuate ABA signal transduction, whereas in the presence of ABA, protein accumulation of MYB96 is elevated, because MIEL1 is degraded by 26S proteasome activity. Consistent with an inhibitory role of MIEL1 in ABA signalling, 35S:*MIEL1-MYC* transgenic seeds were hyposensitive to ABA, while *miel1*-deficient mutant seeds exhibited increased sensitivity to ABA during the germination process.

The MIEL1-MYB96 module is important for regulating ABA-dependent seed germination. While MYB30, another regulatory target of MIEL1, also plays a role in seed germination, its function is most likely independent of MIEL1. Compared with the promotive role of MIEL1 in seed germination, MYB30 also stimulates seed germination in the presence of ABA^57^. The MYB30-deficient *myb30-1* mutant seeds are hypersensitive, whereas *MYB30*-overexpressing transgenic seeds exhibit reduced sensitivity to ABA^57^, which do not suggest a role for MYB30 degradation by MIEL1 during seed germination. In support of this, genetic analysis revealed that *MYB96* is epistatic to *MIEL1*, with respect to the regulation of ABA-dependent seed germination (Fig. 5).

The *MIEL1* gene is also regulated by light, in addition to ABA. Furthermore, it is strongly and specifically expressed in micropylar endosperm, which plays a decisive role in endosperm weakening and thus seed germination^37^,^38^. These observations raise the possibility that MIEL1 might be a crucial molecular component of seed germination process and that MYB96 may not be a sole target of MIEL1 in seeds. For instance, the ELONGATED HYPOCOTYL 5 (HY5) bZIP transcription factor is a plausible candidate, which mediates not only light signalling but also ABA response^58^. HY5 is responsible for the activation of many light-regulated genes and also regulates ABA sensitivity by activating *ABI5* and ABI5-regulated *LATE EMBRYOGENESIS-ABUNDANT* genes in seeds^58^. Consistently, the *hy5* mutants exhibit reduced sensitivity to ABA during seed germination^58^,^59^, and ABA enhances light responses and inhibits hypocotyl elongation in early seedlings^58^,^59^. Although further works are required, MIEL1 might act as an integrator of light and ABA signalling to coordinate proper timing to germinate.

Molecular actions of MIEL1 somewhat differ depending on the plant developmental stages. While MIEL1 primarily acts via MYB96 in the control of seed germination, our data suggest that it may act in crosstalk of ABA and defence signalling during the vegetative stages by promoting protein turnover of both MYB96 and MYB30. On one hand, MIEL1 attenuates defence responses by promoting MYB30 degradation under normal growth condition, but its expression is suppressed upon the exposure to pathogen attack to allow MYB30 accumulation and hypersensitive responses^34^. Notably, the MYB30-mediated defence responses were also stimulated by ABA, presumably due to ABA-dependent MIEL1 degradation. On the other hand, MIEL1 also shapes the MYB96 activity in parallel with the MYB30 pathway. Accumulation of MYB96 levels was enhanced by both ABA treatment and pathogen infection (Fig. 6c). Consistently, we found that MYB96 target genes that are known to be regulated by ABA^15^,^16^,^40^ were also activated in response to pathogen infection.

On the basis of these results, we suggest that MIEL1 may possibly coordinate ABA and defence signalling. It has been proposed that ABA-mediated stress responses are closely associated with defence responses^60^. Abiotic stress can trigger defence responses to prime upcoming pathogen attacks^61^, and pathogen invasion also induces proper defence responses, as well as abiotic stress responses to ensure plant fitness and adaptation to multiple ambient stresses. MIEL1 may play a role in this crosstalk, contributing to the response to both ABA and biotic stresses to establish the efficient protective responses.

## Methods

### Plant materials and growth conditions

*Arabidopsis thaliana* (Columbia-0 ecotype) was used for all experiments unless otherwise specified. Plants were grown under long-day conditions (LDs; 16-h light/8-h dark cycles) with cool white fluorescent light (100 μmol photons m^−2^ s^−1^) at 23 °C. The *myb96-ox* and *myb96-1* mutants (GABI_120B05) were previously reported^15^,^16^,^17^. The *miel1-1* (SALK_097638) and *miel1-2* (SALK_041369) mutants were isolated from a T-DNA insertional mutant pool deposited in the *Arabidopsis* Biological Resource Center (https://abrc.osu.edu/).

To produce transgenic plants overexpressing the *MIEL1* gene, a full-length cDNA was amplified with the primer pair (F: 5′-GAGGCGCGCCATGGAAGCTTCACCCAATG /R: 5′-GAGACGTCTGTTGAGGAAGAACAGGAGGC) and then subcloned into the binary pBA002 vector under the control of the cauliflower mosaic virus 35S promoter. *Agrobacterium tumefaciens*-mediated *Arabidopsis* transformation was then performed.

### Seed germination assays

All genotypes were grown at 23 °C under LDs, and seeds were collected at the same time. Harvested seeds were dried at room temperature at least 1 month before germination assays. For seed germination assays, 40–50 seeds for each line were sterilized and plated on MS medium (half-strength MS salts, 0.05% MES, pH 5.7 and 1% agar) supplemented with various concentrations of ABA (0, 1, 3 μM). Plates were stratified in darkness for 3 days at 4 °C and transferred to a culture room set at 23 °C with a 16-h light/8-h dark cycle. Germination was scored at the indicated time points by counting the frequency of radicle emergence from the seed coat and endosperm. For each germination assay, biological triplicates were performed.

### Quantitative real-time RT–PCR analysis

Total RNA was extracted using TRI reagent (TAKARA Bio, Singa, Japan) according to the manufacturer's recommendations. RT was performed using Moloney Murine Leukaemia Virus reverse transcriptase (Dr Protein, Seoul, South Korea) with oligo (dT18) to synthesize first-strand cDNA from 2 μg of total RNA. Total RNA samples were pretreated with an RNAse-free DNAse. cDNAs were diluted to 100 μl with TE buffer, and 1 μl of diluted cDNA was used for PCR amplification.

Quantitative RT–PCR reactions were performed in 96-well blocks using the Step-One Plus Real-Time PCR System (Applied Biosystems). The PCR primers used are listed in Supplementary Table 1. The values for each set of primers were normalized relative to the *EUKARYOTIC TRANSLATION INITIATION FACTOR 4A1* (*eIF4A*) gene (At3g13920). All RT–qPCR reactions were performed with biological triplicates using total RNA samples extracted from the three independent replicate samples. The comparative ΔΔ*C*_T_ method was employed to evaluate relative quantities of each amplified product in the samples. The threshold cycle (*C*_T_) was automatically determined for each reaction with the analysis software set using default parameters. The specificity of the RT–qPCR reactions was determined by melt curve analysis of the amplified products using the standard method employed by the software.

### Y2H assays

Y2H assays were performed using the BD Matchmaker system (Clontech, Mountain View, CA, USA). The pGADT7 vector was used for the GAL4 AD fusion, and the pGBKT7 vector was used for GAL4 BD fusion. The yeast strain AH109 harbouring the *LacZ* and *His* reporter genes was used. PCR products were subcloned into the pGBKT7 and pGADT7 vectors. The expression constructs were co-transformed into yeast AH109 cells and transformed cells were selected by growth on SD/-Leu/-Trp medium and SD/-Leu/-Trp/-His/-Ade. Interactions between proteins were analysed by measuring the β-galactosidase activity using *o*-nitrophenyl-β-D-galactopyranoside as a substrate.

### BiFC assays

BiFC assays were performed as described previously^62^. In brief, the *MYB96* gene was fused in-frame to the 5′ end of a gene sequence encoding the C-terminal half of EYFP in the pSATN-cEYFP-C1 vector (E3082). The *MIEL1* cDNA sequence was fused in-frame to the 5′ end of a gene sequence encoding the N-terminal half of EYFP in the pSATN-nEYFP-C1 vector (E3081). Expression constructs were co-transformed into *Arabidopsis* protoplasts. Expression of the fusion constructs was monitored by fluorescence microscopy using a Zeiss LSM510 confocal microscope (Carl Zeiss, Jena, Germany).

### Co-IP assays

*A. tumefaciens* cells containing MIEL1-MYC and MYB96-GFP constructs were injected to 3-week-old *N. benthamiana* leaves. Tobacco leaves were homogenized in protein extraction buffer (25 mM Tris-HCl, pH 7.5, 150 mM NaCl, 5% glycerol, 0.05% Nonidet P-40, 2.5 mM EDTA, 1 mM phenylmethylsulfonyl fluoride and 1 × complete cocktail of protease inhibitors). After protein extraction, anti-MYC antibodies (05-724, Millipore, Billerica, MA, USA) coupled to Protein-A sepharose beads (Sigma-Aldrich, St Louis, MO, USA) were mixed and incubated for 4 h at 4 °C. The precipitated samples were washed at least four times with the protein extraction buffer and then eluted by 1 × SDS–polyacrylamide gel electrophoresis (PAGE) loading buffer to subject to SDS–PAGE with anti-MYC (1:2000 dilution; Millipore) or anti-GFP antibodies (1:1,000 dilution; sc-9996, Santa Cruz Biotech., Dallas, Texas, USA). The original gel images are shown in Supplementary Fig. 20.

### Treatment of seedlings with ABA

For treatment with ABA, 2-week-old seedlings grown under LDs were transferred to half-strength MS-liquid medium supplemented with 20 μM (+)-*cis*,*trans*-ABA (L06278; Alfa Aesar, Ward Hill, MA, USA).

### Immunoblot analysis

Harvested plant materials were ground in liquid nitrogen and total cellular extracts were suspended in SDS–PAGE sample loading buffer. The protein samples were then analysed by SDS/PAGE (10% gels) and blotted onto polyvinylidene difluoride membranes (Roche, Indianapolis, IN, USA). Epitope-tagged proteins were immunologically detected using anti-MYC (1:2,000 dilution; Millipore) or anti-Ub antibodies (1:1,000 dilution; sc-0817, Santa Cruz Biotech., Dallas, Texas, USA). Bands from at least three independent blots were quantified using Image J software (http://imagej.nih.gov) and averaged. The original gel images are shown in Supplementary Fig. 20.

### Ubiquitination assays

Plant materials were pretreated with 50 μM MG132 (Calbiochem, Darmstadt, Germany) for 24 h and used for nuclear extraction. Nuclear extracts and anti-MYC antibodies coupled to Protein-A sepharose beads (Sigma-Aldrich) were mixed in extraction buffer (20 mM Tris, pH 7.4, 100 mM sodium chloride, 0.5% Nonidet P-40, 0.5 mM EDTA, 0.5 mM PMSF and protease inhibitor cocktail) containing 50 μM MG132 and incubated for 2 h at 23 °C. The beads were recovered by centrifugation and washed with extraction buffer. The bound proteins were eluted with 1 × SDS–PAGE loading buffer and subjected to SDS–PAGE. Immunological analysis was performed using anti-Ub (1:1,000 dilution; Santa Cruz Biotech.) and anti-MYC antibodies (1:2,000 dilution; Millipore).

### Pathogen infection

Four-week-old plants grown in soil under LDs were used for pathogen infection. The avirulent *P. syringae* pv. *tomato* DC3000*/avrRpm1* (*Pst* DC3000/*avrRpm1*) strain were cultured for 24 h at 27 °C in Luria-Bertani medium supplemented with rifampicin (50 mg l^−1^). A bacterial cell suspension was prepared at OD_600_=0.02 in 10 mM MgCl_2_ and pressure-infiltrated into the fourth and fifth rosette leaves. The inoculated plants were transferred to a growth chamber set at 23 °C with relative humidity of 80% and further grown for 2 days under LDs.

### Data availability

The authors declare that all data supporting the findings of this study are available in the manuscript and its supplementary files or are available from the corresponding author upon reasonable request.

## Additional information

**How to cite this article:** Lee, H. G. & Seo, P. J. The *Arabidopsis* MIEL1 E3 ligase negatively regulates ABA signalling by promoting protein turnover of MYB96. *Nat. Commun.* 7:12525 doi: 10.1038/ncomms12525 (2016).

## Supplementary Material

Supplementary Information Supplementary Figures 1 - 20 and Supplementary Table 1
