# Supplementary material for: The Arabidopsis MIEL1 E3 ligase negatively regulates ABA signalling by promoting protein turnover of MYB96
Source: Nat Commun. 2016 Sep 12;7:12525. doi: 10.1038/ncomms12525 (PMC5027273; doi:10.1038/ncomms12525)
Supplement: Supplementary Information — Supplementary Figures 1 - 20 and Supplementary Table 1 [file ncomms12525-s1.pdf]

Supplementary Figures

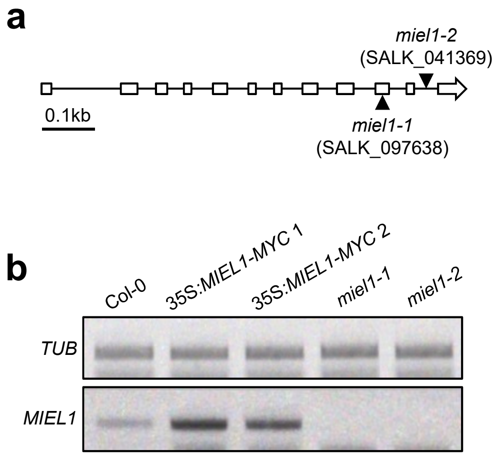

Supplementary Figure 1. *MIEL1* expression in *miel1* mutant and 35S:*MIEL1-MYC* transgenic plants.

(**a**) Mapping of the T-DNA insertion sites of *miel1* mutants. White arrows indicate exons. Black arrowheads indicate T-DNA insertion sites.

(**b**) Transcript accumulation of *MIEL1* in *miel1* mutant and 35S:*MIEL1-MYC* transgenic plants. Ten-day-old seedlings grown under long-day conditions (LDs) were harvested for total RNA isolation. Transcript accumulation was analyzed by semi-quantitative RT-PCR. The *TUBULIN BETA CHAIN 2* (*TUB*) gene (At5g62690) was used as an internal control.

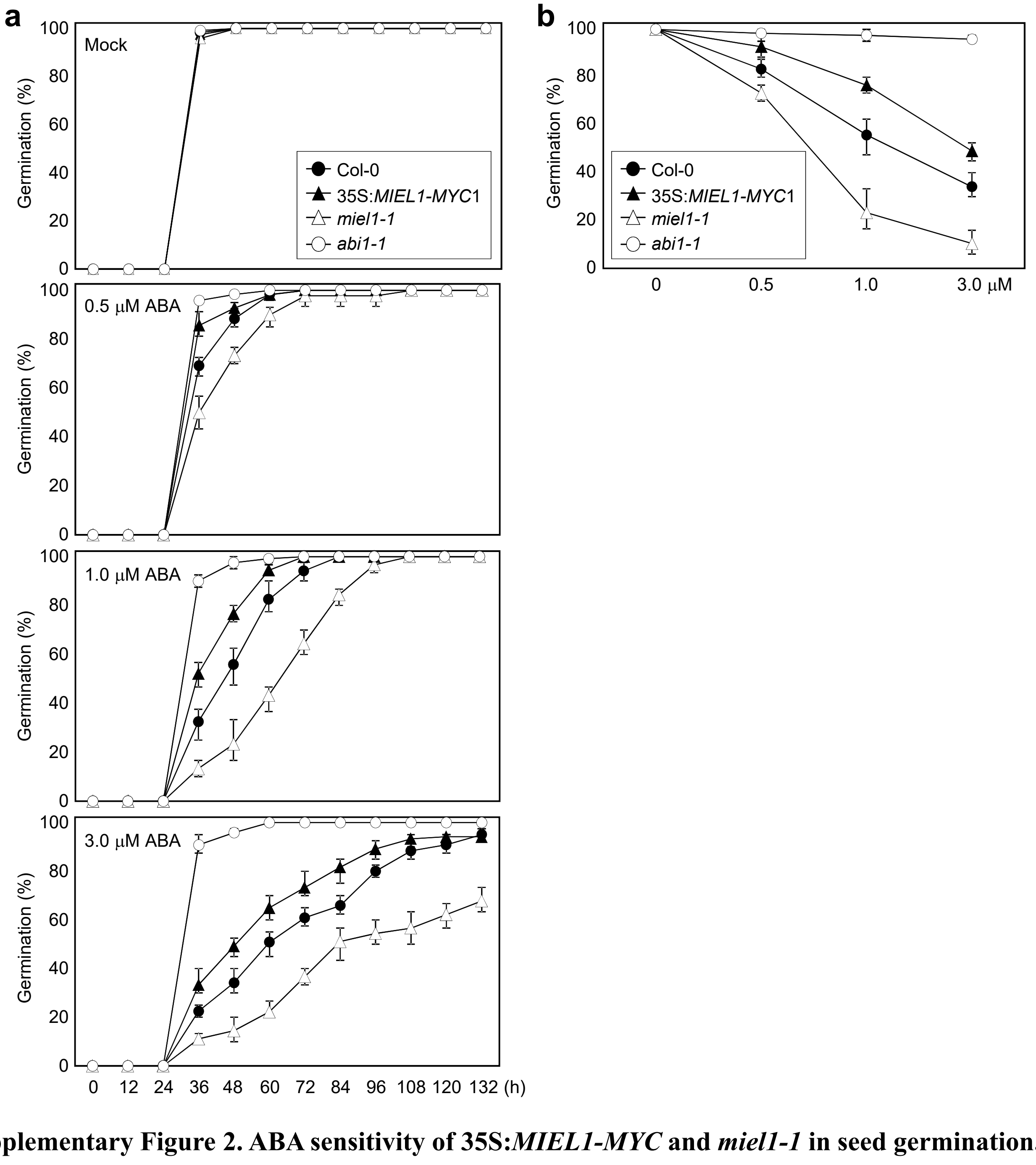

Supplementary Figure 2. ABA sensitivity of 35S:*MIEL1-MYC* and *miel1-1* in seed germination.

(**a**) Germination percentage. Seed germination percentage of the indicated genotypes grown on different concentrations of ABA was quantified after the end of stratification. Radicle emergence was used as a morphological marker for germination. At least 40 seeds per genotype were measured in each replicate. Biological triplicates were averaged. Bars indicate the standard error of the mean. The *abi1-1* mutant was included as a positive control.

(**b**) ABA dose-response assay. Seeds were germinated on an increasing concentration of ABA. Germination percentages were scored 2 days after cold stratification. Biological triplicates were averaged. Bars indicate the standard error of the mean.

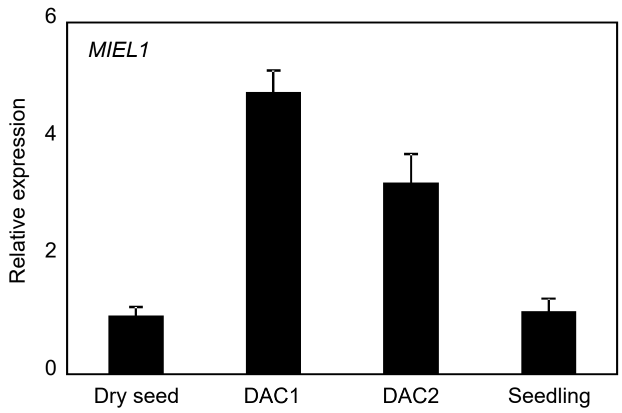

Supplementary Figure 3. Transcript accumulation of *MIEL1* in dry seeds.

Wild-type seeds were kept in darkness at 4°C for 72 h (cold stratification) and transferred to LDs at 22°C for germination. Transcript accumulation was analyzed by quantitative real-time RT-PCR (RT-qPCR). The *EUKARYOTIC TRANSLATION INITIATION FACTOR 4A1* (*eIF4a*) gene (At3g13920) was used as an internal control. Biological triplicates were averaged. Bars indicate the standard error of the mean. Seven-day-old seedlings were included as a comparison. DAC, days after cold stratification.

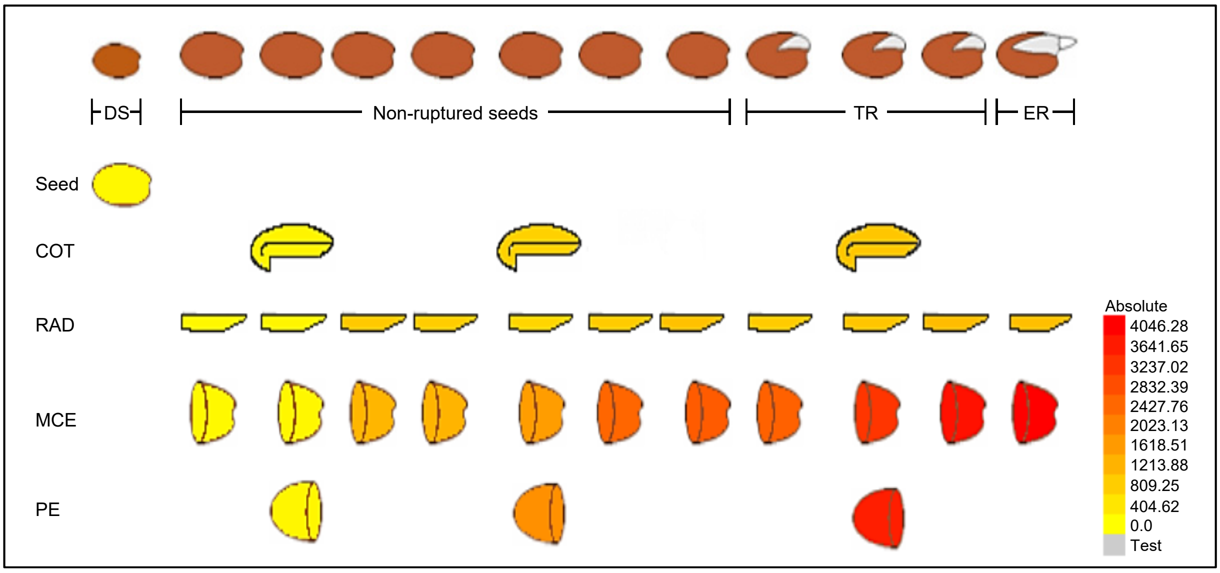

Supplementary Figure 4. Expression of *MIEL1* during seed germination.
The Nottingham Seed eFP Browser (http://ssbvseed01.nottingham.ac.uk) provides spatial and temporal expression of *Arabidopsis* genes in seeds during germination process. The color key in the bottom right-hand corner is for colors in the heat map. DS, dry seed; TR, testa rupture; ER, endosperm rupture; COT, cotyledon; RAD, radicle; MCE, micropylar and chalazal endosperm; PE, peripheral endosperm.

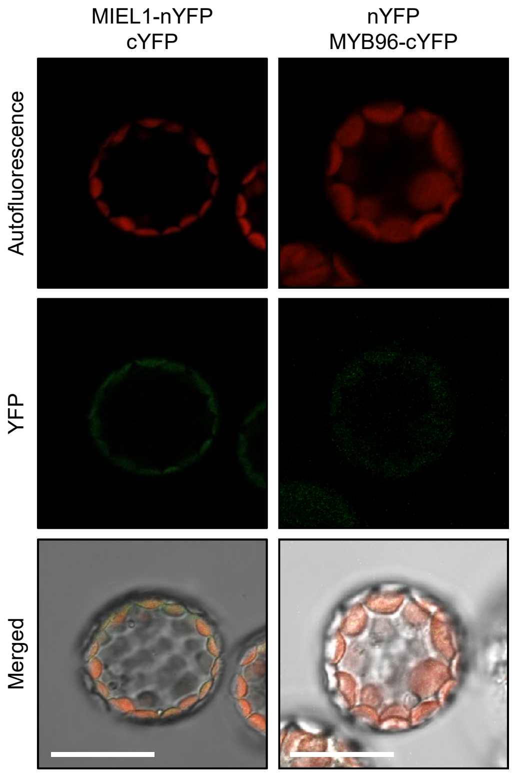

Supplementary Figure 5. Negative controls of BiFC assays.
MIEL1 and MYB96 fused with nYFP or cYFP and the empty vectors were transiently co-expressed in *Arabidopsis* protoplasts as negative controls. No YFP signals were detected. Chloroplasts appear in red. Scale bars = 20 μM

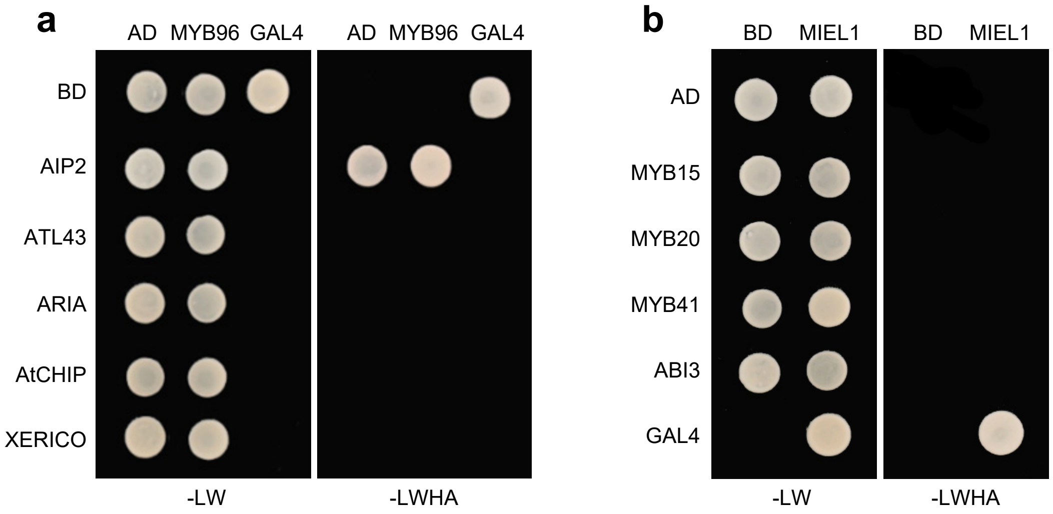

Supplementary Figure 6. Yeast-two-hybrid (Y2H) assays.

(**a**) Interactions of MYB96 with E3 ligases involved in ABA signaling. Y2H assays were performed with the E3 ligase proteins fused with the DNA binding domain (BD) of GAL4 and MYB96 fused with the transcriptional activation domain (AD) of GAL4 for analysis of their interactions.

(**b**) Interactions of MIEL1 with transcriptional regulators of ABA signaling. The GAL4 BD-MIEL1 fusion was coexpressed with transcription factors fused with GAL4 AD in yeast cells. In (**a**) and (**b**), interactions were examined by cell growth on selective media. -LWHA indicates Leu, Trp, His, and Ade drop-out plates. -LW indicates Leu and Trp drop-out plates. GAL4 was used as a positive control.

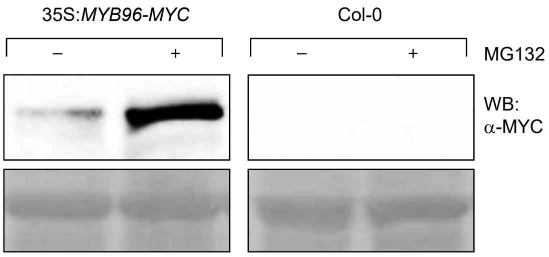

Supplementary Figure 7. Effects of MG132 on MYB96 stability in seedlings.

Two-week-old 35S:*MYB96-MY*C transgenic plants were incubated in MS-liquid medium supplemented with or without 50 μM MG132 for 24 h. MYB96 proteins were detected immunologically using an anti-MYC antibody. Wild-type samples were included to show specificity of the antibody. WB, Western blot analysis.

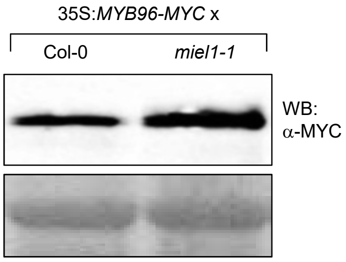

Supplementary Figure 8. MYB96 accumulation in *miel1-1* background.

Two-week-old plants were harvested for total protein isolation. MYB96 proteins were detected immunologically using an anti-MYC antibody. WB, Western blot analysis.

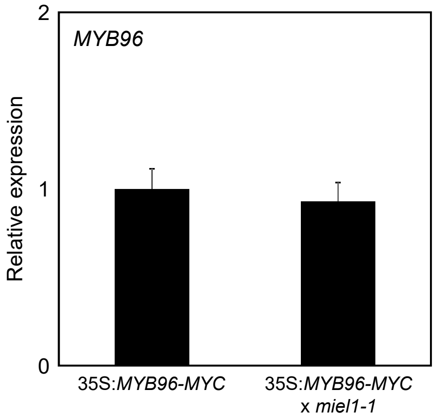

Supplementary Figure 9. Expression of *MYB96* in 35S:*MYB96-MYC* and 35S:*MYB96-MYC*x*miel1-1*. Two-week-old plants grown under LDs were harvested for total RNA isolation. Transcript accumulation was analyzed by RT-qPCR. Biological triplicates were averaged. Bars indicate standard error of the mean.

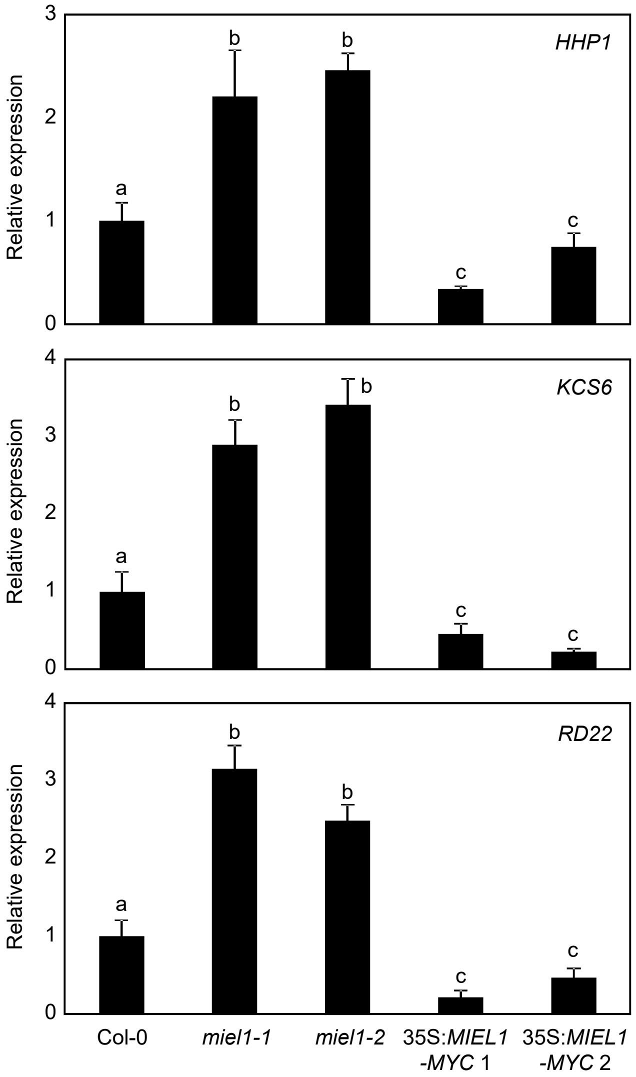

Supplementary Figure 10. Expression of ABA-inducible genes regulated by MYB96 in *miel1* mutant and 35S:*MIEL1-MYC* transgenic plants.

Two-week-old plants grown under LD conditions were used to analyze transcript accumulation. The *eIF4a* gene was used as an internal control. Biological triplicates were averaged. Bars indicate the standard error of the mean. Different letters represent a significant difference at $P < 0.05$ (one-way anova with Fisher's *post hoc* test).

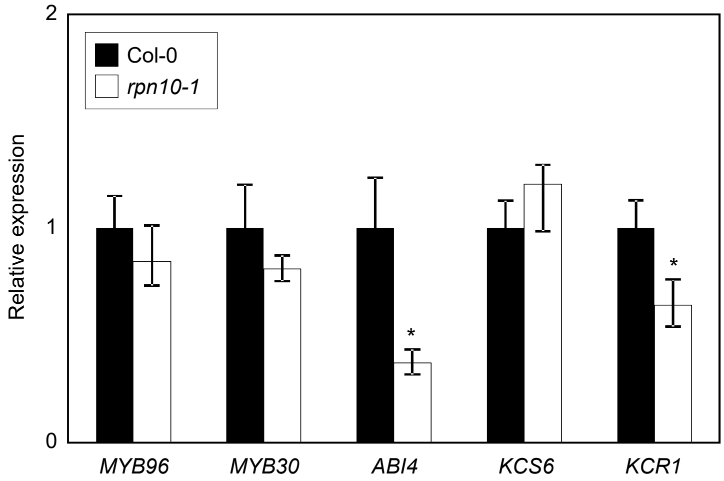

Supplementary Figure 11. Expression of MYB96 target genes in *rpn10-1*.

Two-week-old plants grown under LD conditions were used to analyze transcript accumulation. The *eIF4a* gene was used as an internal control. Biological triplicates were averaged. Bars indicate the standard error of the mean. Statistically significant differences between wild-type and mutant plants are indicated by asterisks (**P*<0.05, Student's *t*-test).

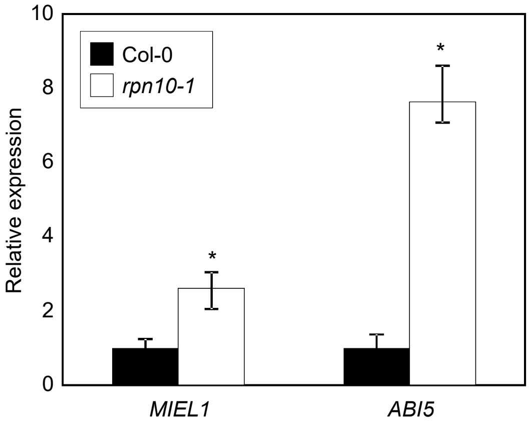

Supplementary Figure 12. Transcript accumulation of *MIEL1* in *rpn10-1*.

Dry seeds were used to analyze transcript accumulation. The *eIF4a* gene was used as an internal control. Biological triplicates were averaged. Bars indicate the standard error of the mean. Statistically significant differences between wild-type and mutant plants are indicated by asterisks (**P*<0.05, Student's *t*-test). Note that the *ABI5* gene was included as a positive control.

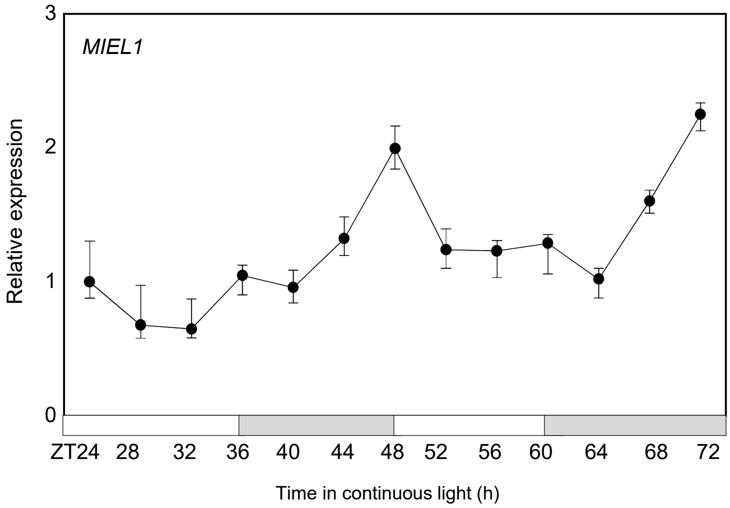

Supplementary Figure 13. Circadian expression of *MIEL1*.
Seedlings grown under neutral day conditions (12-h light/12-h dark) for 10 days were transferred to continuous light conditions at zeitgeber time 0 (ZT0). Whole seedlings were harvested from ZT24 to ZT72. Transcript levels were determined by RT-qPCR. Biological triplicates were averaged. Bars indicate the standard error of the mean.

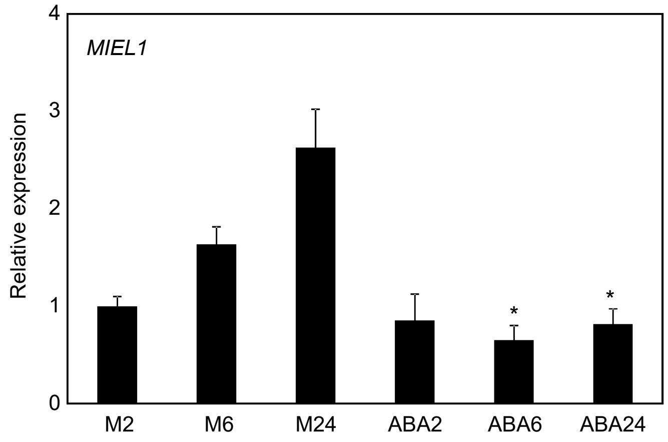

Supplementary Figure 14. Effects of ABA on transcript accumulation of *MIEL1* in seedlings. Two-week-old plants grown under LD conditions were transferred to MS-liquid medium supplemented with or without 20 μM ABA and incubated for up to 24 h. Transcript accumulation was analyzed by RT-qPCR. The *eIF4a* gene was used as an internal control. Biological triplicates were averaged. Bars indicate the standard error of the mean. Statistically significant differences between mock (M) and ABA-treated samples at corresponding time points are indicated by asterisks (**P*<0.05, Student's *t*-test).

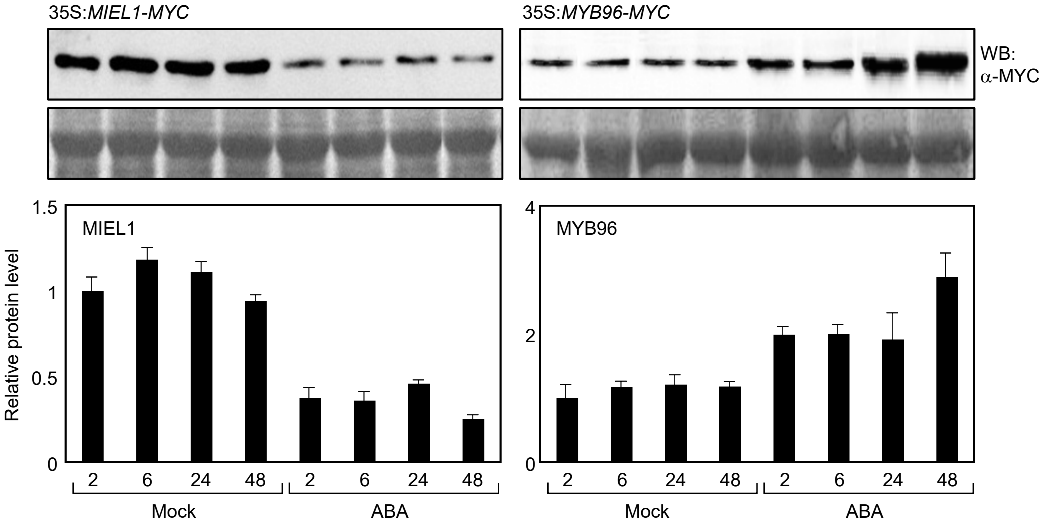

Supplementary Figure 15. Protein abundance of MIEL1 and MYB96 in the presence of ABA. Two-week-old plants were incubated in MS-liquid medium supplemented with or without 20 μM ABA for the indicated time periods (h). Proteins were detected immunologically using an anti-MYC antibody. Bands from three independent blots were quantified using Image J software and averaged (bottom panels). Bars indicate the standard error of the mean. WB, Western blot analysis.

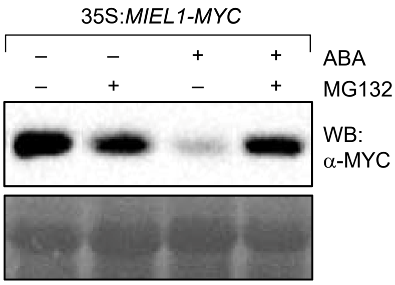

Supplementary Figure 16. Effects of MG132 on ABA-dependent MIEL1 degradation.
Two-week-old plants were treated with 20 μM ABA and/or 50 μM MG132 for 24 h. MIEL1 proteins were detected immunologically using an anti-MYC antibody. WB, Western blot analysis.

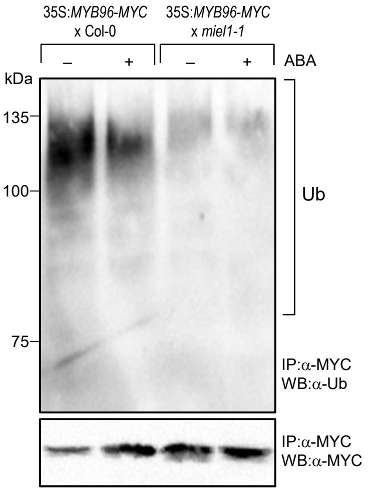

Supplementary Figure 17. Ubiquitination of MYB96 by MIEL1 in seedlings.
Two-week-old plants were pretreated with 50 μM MG132 for 24 h before treatment with 20 μM ABA for 6 h. Total protein extracts were immunoprecipitated using anti-MYC antibodies coupled to agarose beads and were subjected to Western blot analysis using anti-ubiquitin (top panel) and anti-MYC (bottom panel) antibodies. Ub, ubiquitinated MYB96 proteins; IP, immunoprecipitation; WB, Western blot analysis.

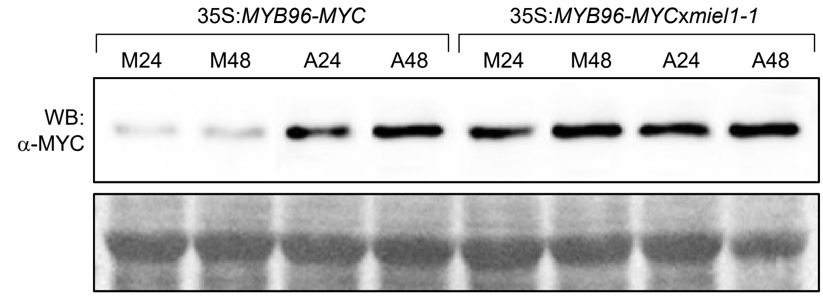

Supplementary Figure 18. Effects of ABA on MYB96 stability in *miel1-1* seedlings.
Two-week-old plants were incubated in MS-liquid medium supplemented with or without 20 μM ABA for the indicated time periods (h). MYB96 proteins were detected immunologically using an anti-MYC antibody. M, mock; A, ABA; WB, Western blot analysis.

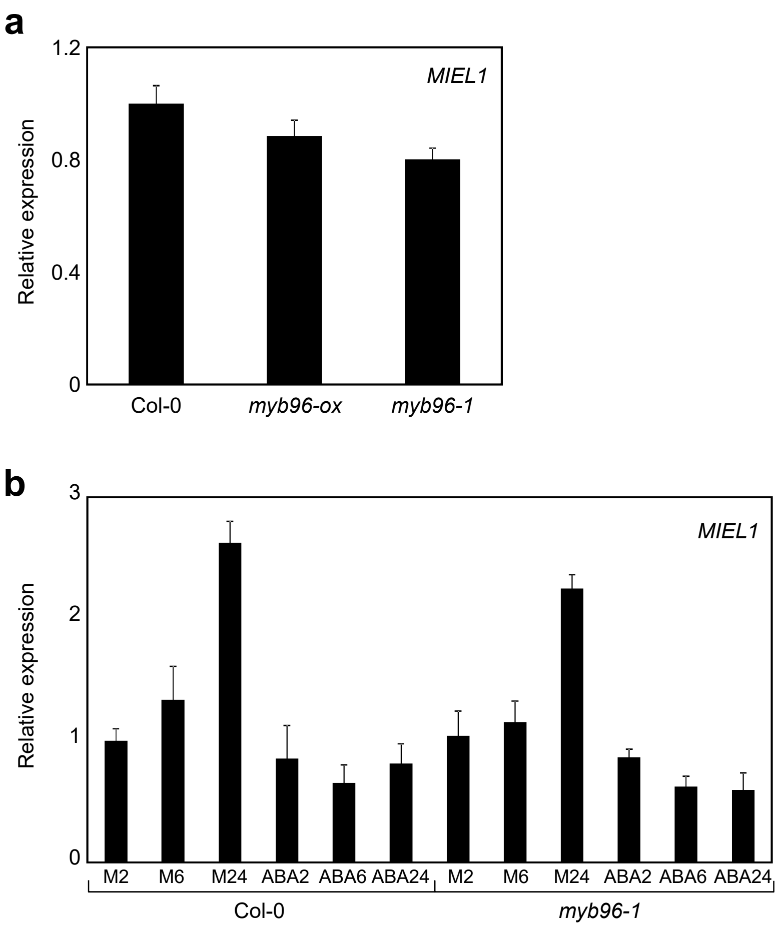

Supplementary Figure 19. Feedback regulation of *MIEL1* by MYB96.

(**a**) Expression of *MIEL1* in *myb96-ox* and *myb96-1* mutants. Two-week-old plants grown under LDs were harvested to analyze transcript accumulation. Biological triplicates were averaged. Bars indicate the standard error of the mean.

(**b**) Effects of ABA on *MIEL1* expression in *myb96-1*. Two-week-old plants grown under LDs were transferred to half-strength MS-liquid medium supplemented with or without 20 μM ABA for the indicated time period (h). Transcript accumulation was analyzed by RT-qPCR. Biological triplicates were averaged. Bars indicate the standard error of the mean. M, mock.

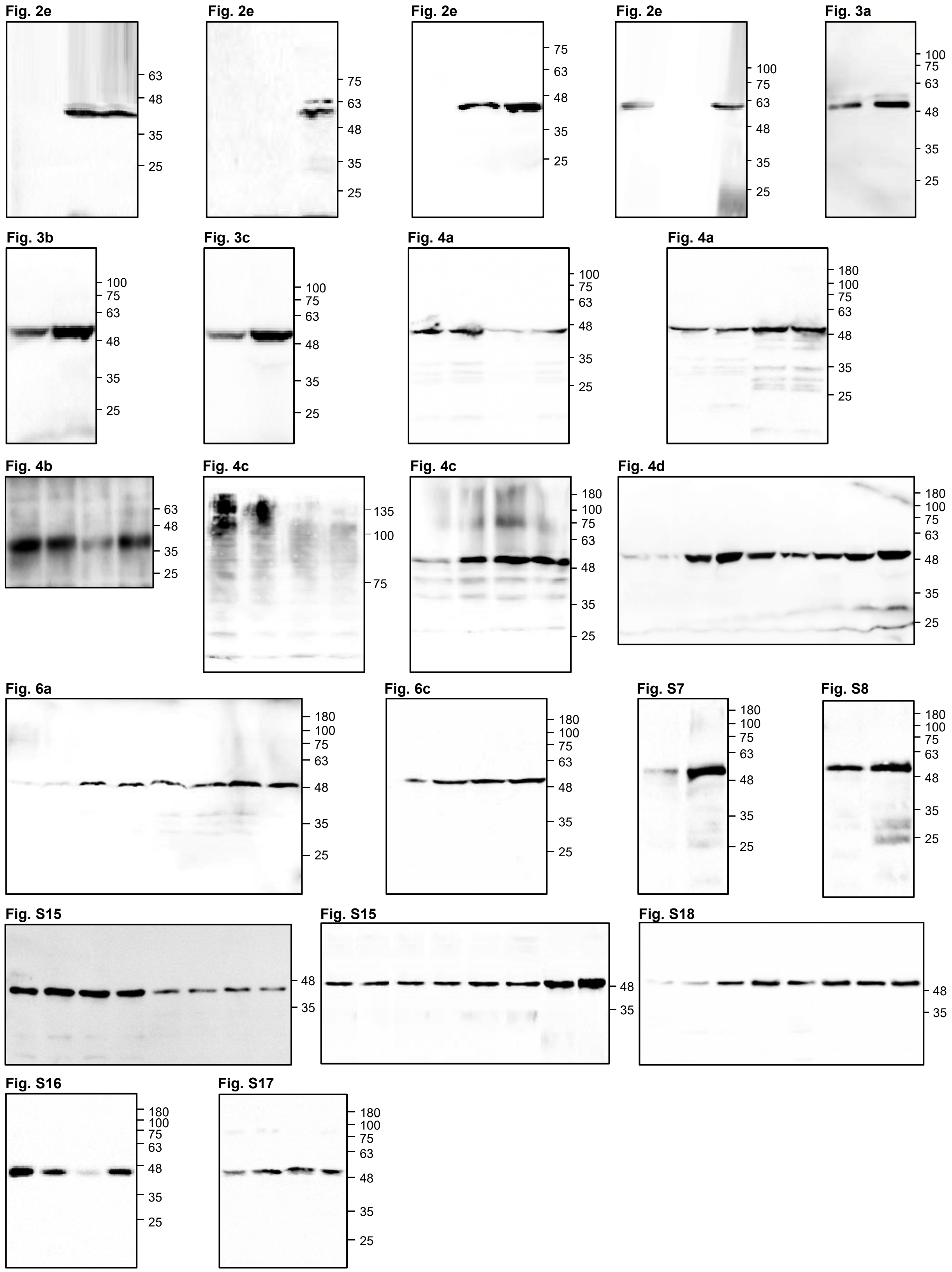

Supplementary Figure 20. The original full immunoblot images utilized in this study.

Supplementary Tables

Primer	Usage	Sequence
eIF4a-F	RT-qPCR	5′- TGACCACACAGTCTCTGCAA
eIF4a-R	RT-qPCR	5′- ACCAGGGAGACTTGTTGGAC
MYB96-F	RT-qPCR	5′- TGCAGTCTCGGAAGAAGGTG
MYB96-R	RT-qPCR	5′- CATCTCGTGGCTTTGCTCAT
MYB30-F	RT-qPCR	5′- ACTTGACCGTTCCTCGCTCT
MYB30-R	RT-qPCR	5′- GTAATGATTGGTGCAACGGC
MIEL1-F	RT-qPCR	5′- GCGTTGAGAATTCAATGCGT
MIEL1-R	RT-qPCR	5′- TGGCTTCGATCTCTTCATCG
ABI4-F	RT-qPCR	5′- ATCCTCAATCCGATTCCACC
ABI4-R	RT-qPCR	5′- ATTTGCCCCAGCTTCTTTGT
ABI5-F	RT-qPCR	5′- GGCGCAAGCGAGACATAAT
ABI5-R	RT-qPCR	5′- CCCTCGCCTCCATTGTTATT
CER2-F	RT-qPCR	5′- AGATTCGGTTGGCGAGTATTGGTTA
CER2-R	RT-qPCR	5′- GCATACAAAATATCAACCTCCGAGA
CER10-F	RT-qPCR	5′- TGACTCTTCCCGTGACTCCT
CER10-R	RT-qPCR	5′- CGAAGAAGAAGAGTGTGCGGTA
HHP1-F	RT-qPCR	5′- GCAAGCAGCATTTGTCACCT
HHP1-R	RT-qPCR	5′- TGCCTGCGAGGTAGATGAAG
RD22-F	RT-qPCR	5′- AGGAGCAAACCCTTTCGTGT
RD22-R	RT-qPCR	5′- CGTTTCAACGTCTCCGAAAA
KCS6-F	RT-qPCR	5′- TATTGTCGCCGTTGAGCTTC
KCS6-R	RT-qPCR	5′- TTCCATGAAAGTTGCGAAGG
SID2-F	RT-qPCR	5′- TCTGCAGTGAAGCTTTGGCT
SID2-R	RT-qPCR	5′- GGTCGTCTTTCGGACTGGTT
KCR1-F	RT-qPCR	5′- CAAATCACAACCCACTTGGC
KCR1-R	RT-qPCR	5′- AGGTTTTTGGATGGTCGGAG
TUB-F	RT-PCR	5′- CTCAAGAGGTTCTCAGCAGTA
TUB-R	RT-PCR	5′- TCACCTTCTTCATCCGCAGTT
MIEL1-F(SALK_097638)	RT-PCR	5′- GATCTTGTTATGCGGTTGGTC
MIEL1-R(SALK_097638)	RT-PCR	5′- TAATCCTCGCATTTCCAACAC
MIEL1-F(SALK_041369)	RT-PCR	5′- CTTCCATTGCAAGAAGTGTG
MIEL1-R(SALK_041369)	RT-PCR	5′- CTCGTGTGTTGTATGATCTGC
MIEL1-F(E3081/2)	Subcloning	5′- GACCCGGGAATGGAAGCTTCACCCAATG
MIEL1-R(E3081/2)	Subcloning	5′- GAGGATCCTCATTGAGGAAGAACAGGAGG
MIEL1-F(E3083/4)	Subcloning	5′- GACCCGGGATGGAAGCTTCACCCAATG
MIEL1-R(E3083/4)	Subcloning	5′- GAGGATCCTTTGAGGAAGAACAGGAGGC
MYB96-F(E3081/2)	Subcloning	5′- GACTCGAGCTATGGGAAGACCACCTTGC
MYB96-R(E3081/2)	Subcloning	5′- GAGAATTCTCTAGAACATCCCTTCTTGTCCTG
MYB96-F(E3083/4)	Subcloning	5′- GACTCGAGATGGGAAGACCACCTTGC
MYB96-R(E3083/4)	Subcloning	5′- GAGAATTCGAACATCCCTTCTTGTCCTG
MYB96-F(pGADT7)	Subcloning	5′- GACATATGATGGGAAGACCACCTTGC
MYB96-R(pGADT7)	Subcloning	5′- GAGAATTCCTAGAACATCCCTTCTTGTCCTG
MYB96^{N}-F(pGADT7)	Subcloning	5′- GACATATGATGGGAAGACCACCTTGC
MYB96^{N}-R(pGADT7)	Subcloning	5′- GAGAATTCCTAGCTTTGATGGTTCTTTTGTG
MYB96$^{\Delta N}$-F(pGADT7)	Subcloning	5′- GACCATGGAGACTAACAAAGGTCAATGGGAAAG
MYB96$^{\Delta N}$-R(pGADT7)	Subcloning	5′- GAGAATTCCTAGAACATCCCTTCTTGTCCTG
MYB96^{M}-F(pGADT7)	Subcloning	5′- GACCATGGAGACTAACAAAGGTCAATGGGAAAG
MYB96^{M}-R(pGADT7)	Subcloning	5′- GACCCGGGCTAGTCTTTCATGAAATCTTGAAGC
MYB96^{C}-F(pGADT7)	Subcloning	5′- GACCATGGAGACACCCAAGGCTTTAACTTTATC
MYB96^{C}-R(pGADT7)	Subcloning	5′- GAGAATTCCTAGAACATCCCTTCTTGTCCTG
MIEL1-F(pGBKT7)	Subcloning	5′- GACATATGATGGAAGCTTCACCCAATG
MIEL1-R(pGBKT7)	Subcloning	5′- GACCCGGGTCATTGAGGAAGAACAGGAGG
MIEL1^{N}-F(pGBKT7)	Subcloning	5′- GACATATGATGGAAGCTTCACCCAATG
MIEL1^{N}-R(pGBKT7)	Subcloning	5′- GACCCGGGTCAGTTGCGCAGACCAAC
MIEL1^{M}-F(pGBKT7)	Subcloning	5′- GACATATGAACCATCGCTGCGTTG
MIEL1^{M}-R(pGBKT7)	Subcloning	5′- GACCCGGGTCAAGACATATCAATCACTGACCTC
MIEL1^{C}-F(pGBKT7)	Subcloning	5′- GACATATGAAAACATGGCAGAGACTCGATG
MIEL1^{C}-R(pGBKT7)	Subcloning	5′- GACCCGGGTCATTGAGGAAGAACAGGAGG

(to be continued)

Primer	Usage	Sequence
AIP2-F(pGBKT7)	Subcloning	5′- GACCATGGATATGGATGCATCGTCTTCACC
AIP2-R(pGBKT7)	Subcloning	5′- GACCCGGGTTAAACGTACATATATTCACCTCCG
ATL43-F(pGBKT7)	Subcloning	5′- GACCATGGATATGCCCGGAATCGCAGT
ATL43-R(pGBKT7)	Subcloning	5′- GACCCGGGTTAAACAATGCTGGAAGCGG
ARIA-F(pGBKT7)	Subcloning	5′- GACCATGGATATGGACCAACAACCGGAGAG
ARIA-R(pGBKT7)	Subcloning	5′- GACCCGGGCTACAACCTCAAGCTTTGCAGG
AtCHIP-F(pGBKT7)	Subcloning	5′- GACCCGGGTATGGTTACAGGCGTGGCTTC
AtCHIP-R(pGBKT7)	Subcloning	5′- GAGGATCCTCAACAACCCATCTTGTAAGCC
XERICO-F(pGBKT7)	Subcloning	5′- GACCATGGATATGGGTCTATCAAGTCTTCCTGG
XERICO-R(pGBKT7)	Subcloning	5′- GACCCGGGTCACCAAACATTAGAAGAAAGCTG
MYB15-F(pGADT7)	Subcloning	5′- GACATATGATGGGAAGAGCTCCATGCTG
MYB15-R(pGADT7)	Subcloning	5′- GACCCGGGCTAGAGCCCGGCTAAGAGATC
MYB20-F(pGADT7)	Subcloning	5′- GACATATGATGGGGAGACAACCATGCTG
MYB20-R(pGADT7)	Subcloning	5′- GACCCGGGTCAAAACAACCCAAAAGTGGAAG
MYB41-F(pGADT7)	Subcloning	5′- GACATATGATGGGAAGATCACCTTGTTGTG
MYB41-R(pGADT7)	Subcloning	5′- GACCCGGGTTAAAACATAAAGTCATCTAAGATGAAATC
ABI3-F(pGADT7)	Subcloning	5′- GACATATGATGAAAAGCTTGCATGTGGC
ABI3-R(pGADT7)	Subcloning	5′- GAATCGATTCATTTAACAGTTTGAGAAGTTGGTG

Supplementary Table 1. Primers used in this study.
RT-qPCR primers were designed using the Primer Express Software installed into the Applied Biosystems 7500 Real-Time PCR System. The sizes of PCR products ranged from 80 to 300 nucleotides in length. F, forward primer; R, reverse primer.
